# Attitudes about Tuberculosis Prevention in the Elimination Phase: A Survey among Physicians in Germany

**DOI:** 10.1371/journal.pone.0112681

**Published:** 2014-11-13

**Authors:** Christian Gutsfeld, Ioana D. Olaru, Oliver Vollrath, Christoph Lange

**Affiliations:** 1 Division of Clinical Infectious Diseases, German Center for Infection Research Tuberculosis Unit, Research Center Borstel, Borstel, Germany; 2 Department of Psychosomatic Medicine, Sachsenklinik, Bad Lausick, Germany; 3 Institute of Medical Informatics and Statistics, University Hospitals Schleswig-Holstein, Campus Kiel, Kiel, Germany; 4 International Health/Infectious Diseases, University of Lübeck, Lübeck, Germany; 5 Department of Internal Medicine, University of Namibia School of Medicine, Windhoek, Namibia; 6 Department of Medicine, Karolinska Institute, Stockholm, Sweden; FIOCRUZ, Brazil

## Abstract

**Background:**

Targeted and stringent measures of tuberculosis prevention are necessary to achieve the goal of tuberculosis elimination in countries of low tuberculosis incidence.

**Methods:**

We ascertained the knowledge about tuberculosis risk factors and stringency of tuberculosis prevention measures by a standardized questionnaire among physicians in Germany involved in the care of individuals from classical risk groups for tuberculosis.

**Results:**

510 physicians responded to the online survey. Among 16 risk factors immunosuppressive therapy, HIV-infection and treatment with TNF-antagonist were thought to be the most important risk factors for the development of tuberculosis in Germany. Exposure to a patient with tuberculosis ranked on the 10^th^ position. In the event of a positive tuberculin-skin-test or interferon-γ release assay only 50%, 40%, 36% and 25% of physicians found that preventive chemotherapy was indicated for individuals undergoing tumor necrosis factor-antagonist therapy, close contacts of tuberculosis patients, HIV-infected individuals and migrants, respectively.

**Conclusions:**

A remarkably low proportion of individuals with latent infection with *Mycobacterium tuberculosis* belonging to classical risk groups for tuberculosis are considered candidates for preventive chemotherapy in Germany. Better knowledge about the risk for tuberculosis in different groups and more stringent and targeted preventive interventions will probably be necessary to achieve tuberculosis elimination in Germany.

## Introduction

Tuberculosis (TB) remains a major global health problem. In 2012, an estimated 8.6 million people developed TB and 1.3 million died from the disease (including 320,000 deaths among HIV-positive people) [1]. However in most of the low-prevalence countries in Western Europe and North America overall notification rates for TB have been declining for the last decades. With a total incidence of 5.3 cases per 100,000 population in 2011, TB has become a rare disease in Germany [2].

Due to the current absence of vaccines for the prevention of TB with a higher efficiency than the *Mycobacterium bovis* Bacille Calmette Guérin (BCG) vaccine, TB control primarily relies on the prevention of transmission of active TB by identification and treatment of patients with active disease. In order to further reduce transmissions rates and ultimately eradicate TB, low incidence countries such as Germany eventually rely on contact investigations by public health services and physicians for active case finding and identification of contacts with latent infection with *Mycobacterium tuberculosis* (LTBI) [3], [4].

In clinical practice, LTBI is defined by the presence of an adaptive immune response to antigens specific for *M. tuberculosis*, ascertained by a positive tuberculin-skin-test (TST) or interferon-γ release assay (IGRA) result, in the absence of active TB [4]. According to the Infection Protection Act (IFSG) in Germany close contacts of TB patients are required to be subject to a TST or IGRA testing by the public health authorities [5]. In case of positive test results national recommendations suggest preventive chemotherapy with isoniazid for a duration of nine months [6]. A contact person with LTBI will usually be referred to a private physician with the recommendation to initiate preventive chemotherapy [7]. In contrast to neighboring Switzerland [8], most of the contacts with LTBI referred to private pulmonologists or general practitioners in Germany by the public health authorities are left untreated. A recent survey in the state of Lower Saxony demonstrated that only 29% of healthy contacts with a positive TST or IGRA test result at the time of contact investigation received preventive chemotherapy [9]. In a large observational cohort study performed by the public health authorities in the city of Hamburg, even under study conditions only 21% of contacts with LTBI received preventive chemotherapy [10]. The low acceptance of preventive chemotherapy contradicts the expenses and efforts by the public health authorities in Germany to identify individuals at risk for the future development of TB.

To develop a basis for improvement of TB prevention we aimed to gain a better insight about the knowledge of physicians working within the German health care system about risk factors for TB and their attitude towards preventive chemotherapy.

## Materials and Methods

To evaluate the knowledge and attitude of physician decision makers in Germany about current methods for the diagnosis of LTBI and preventive chemotherapy, we developed a standardized questionnaire that was initially revised by two independent international reviewers. Feasibility of this survey tool was evaluated in an anonymized pilot study involving distribution of paper questionnaires to 500 physicians. The return rate by mail was 130 questionnaires (26%). Results from the feasibility study were not included in the final analysis. Upon the experience with the pilot survey, it was decided to distribute the questionnaire by email via an internet-link leading to a web-based survey platform (www.surveymonkey.com). Instructions about the study background, design and participation were found in the study invitation and on the web-based survey platform. In February 2012 email invitations were send to physicians from the registries of the German Society for Pulmonology (DGP e.V.), the German working group of private physicians caring for HIV-infected patients (DAGNÄ e.V.), the network for rheumatic diseases (DGRh), the German network of occupational medicine physicians (ArbMedNet), and to TB officers at municipal or regional health care departments in Germany. Physicians were asked to participate in the survey within 30 days of notice. In order to ensure the participants anonymity the gathered results were encrypted by an independent code system.

The web-based questionnaire was composed of 8 main parts with 26 questions and a free commenting section for further remarks:

Part 1: Six single item questions about specific data of participating physicians (age, working area, expertise and patients collective).
Part 2: Three single item questions about past, current and future use and evaluation of diagnostic tools for LTBI such as TST and/or IGRAs.
Part 3: One multiple answer question with the opportunity to select three risk groups among sixteen options to estimate the risk of patients at risk for developing TB if diagnostic tools are positive.
Part 4: Five single item questions concerning risk groups and the implementation of national guidelines towards LTBI (offering testing and treatment to patients at risk).
Part 5: One single item question about favored therapy regimes for patients with LTBI.
Part 6: Nine questions about the attitudes of physicians towards TB prevention in Germany using six-point adjectival scales that included the response categories full and minimal agreement.
Part 7: One single item question about the necessity of improvement towards management of LTBI using a ranking scale in order to prioritize between diagnostics, period of therapy and effectiveness.

Eligible respondents included physicians who were involved in the diagnosis and/or therapy of LTBI. We excluded physicians who had no contact to a suspected case LTBI within the last 12 months.

### Statistical analysis

The collected data consisted only of categorical variables. They were summarized using frequencies and percentages.

The paired sample McNemar Test was used to test the difference of proportions between past and future application of several diagnostic devices by German physicians (sample size N = 510). All tests were two-sided. A difference was considered statistically significant when the p-value was smaller than 0.05. All p-values were adjusted according to Bonferroni.

In addition to the p-values 95% confidence intervals (95% CI) for differences between past and future applications were calculated.

All data were analyzed using SPSS 19.0 for Windows (SPSS Inc., Chicago, Illinois, USA) and BIAS 9.16 for Windows (epsilon-Verlag; Dr. rer. med H. Ackermann, Goethe-University Frankfurt/Main, Germany).

The study was reviewed and approved by the Ethical Board of the University of Lübeck (#14-167). The study was a voluntary survey of German physicians and all data entered and analyzed were anonymous. There were no patients involved and consent from physicians providing data anonymously was not required. We have been in touch with the Ethical Board at the University of Lübeck and have received the written information that the Ethical Board of the University of Lübeck has no reservations against publication of the study results.

## Results

### Characteristics of German physicians participating in the survey

We contacted 1840 pulmonologists and 354 public health officers directly via email requesting participation in the survey. In addition, approximately 1000 rheumatologists, physicians caring for HIV-infected patients and physicians working in occupational medicine were addressed via the DGRh, DAGNÄ and ArbMedNet email registers. 510 physicians responded to the survey (table 1). Only 15.2% (n = 76) of participating physicians were under the age of 40 and 6.0% (n = 30) were older than 60 years of age. More than one third (38.5%/n = 190) of the participating physicians were working in a hospital setting and almost one quarter (23.9%/n = 118) were working in a private practice. One-hundred and thirty-four physicians (26.3%) were employed as TB officers at municipal or regional health care departments and 11.5% (n = 55) of the physicians were working in the field of occupational medicine. Overall, 250 participants (49.0%) were specialized as pulmonologists.

**Table 1:** Characteristics of German physicians participating in the survey.

Physicians data		Frequency	Percent (%)	n
Age	21–30 years	8	1.6	501
	31–40 years	68	13.6	
	41–50 years	205	40.9	
	51–60 years	190	37.9	
	>60 years	30	6.0	
Work Place	Establishedpractitioner	118	23.9	493
	Teaching hospital	99	20.1	
	Non-teaching Hospital	49	10.0	
	University Hospital	42	8.5	
	Other	185	37.5	
Specialty	Internal Medicine	236	49.2	480
	General Practitioners	26	5.4	
	Occupational Medicine	55	11.5	
	Other	163	33.9	
Subspecialisation	Respiratory Medicine	250	49.0	510
	Public HealthMedicine	134	26.3	
	Other	81	15.9	
	none	45	8.8	

### Experience and future intention to use different tests for diagnosing LTBI

The changes of the attitude towards the methods for the diagnosis LTBI are shown in table 2. Physicians intend to use the ELISPOT IGRA (T-Spot.TB test) significantly more often and the TST significantly less often in the future when compared to the past. There is no significant change in the anticipated behavior for the use of the ELISA IGRA (QFT-GIT test) which is already in frequent use in the county.

**Table 2:** Experience and future intention to use different tests for diagnosing latent infection with Mycobacterium tuberculosis.

Diagnostic device	Past	Future	Difference	95% CI fordifference	p-value	p-value adjusted
	p1	p2	p1–p2			
Tuberculin-Skin-Test	57.1%	34.1%	23%	[18.6; 27.3]	<0.001*	<0.001*
IGRA QuantiFeronGold in tube	69.4%	67.7%	1.7%	[–1.2; 4.7]	0.298	1.000
IGRA T-Spot.TB	27.5%	32.2%	–4.7%	[–7.9; −1.5]	0.005*	0.0196*
Other technologies(e.g. flow cytometry)	5.9%	4.5%	1.4%	[–0.2; 2.9]	0.143	0.572

The sample size was N = 510.

p1: percentage of physicians using the corresponding test in the past.

p2: percentage of physicians using the corresponding test in future.

p1–p2: difference of percentages p1 and p2.

95% CI: 95% confidence interval for difference.

p-value: paired sample McNemar Test to test the difference of proportion between past and future application of several diagnostic devices by german physicians.

p-value adjusted: adjusted p-value according to Bonferroni.

The asterisk (*) indicates significant differences.

### Estimated risk by physicians for persons at risk to develop TB

**Figure 1:**
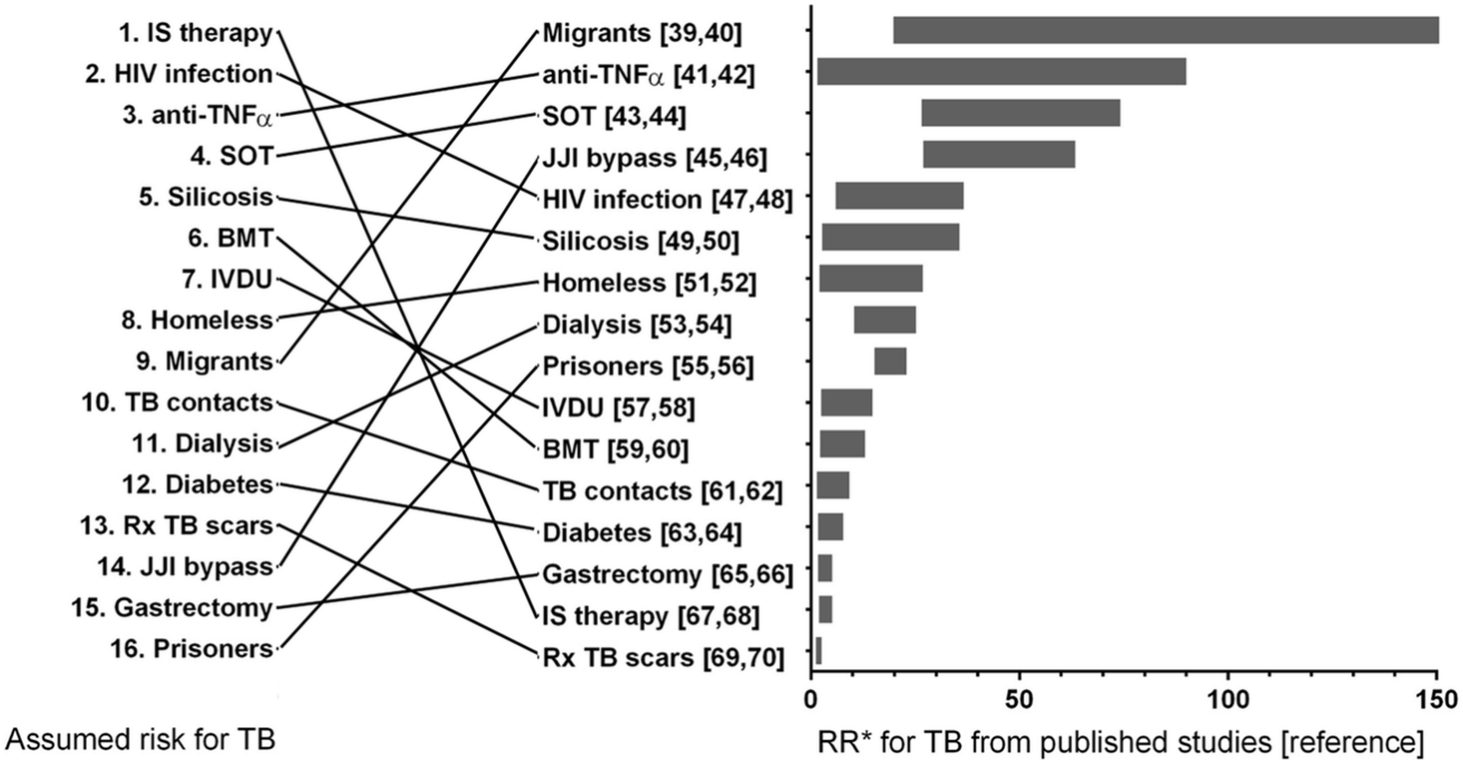
Subjective ranking (1 = highest risk; 16 = lowest risk) of risk groups for the future development of tuberculosis according to German physicians involved in LTBI testing (left) in comparison with the range of reported relative risks (RR) for the development of tuberculosis in the same risk groups according to published studies ranked according to the highest risk reported (right). References are shown in square brackets (max. to min.) [39]–[70]. *Risk is expressed as relative risk for cohort studies or controlled trials, odds ratio for case-control studies and incidence rate ratio when incidence in cases was compared to that in the general population. In the case of migrants the highest value for risk is not plotted on the graph (relative risk of 315.5). TNFα – tumor necrosis factor α, SOT – solid organ transplant, JJI bypass – jejunoileal bypass, IVDU – intravenous drug users, BMT – bone marrow transplant, IS therapy – immunosuppressive therapy, Rx – radiological.

When physicians were asked to prioritize groups with the highest risk for the future development of TB, patients with an immunosuppressive therapy, HIV-seropositive patients and patients with a TNF-antagonist-therapy, were ranked on positions 1–3 among 16 risks groups (figure 1-right). Interestingly, contact persons of patients diagnosed with TB were ranked on position 10/16. Physicians’ attitude of groups at risk for the future development of TB and data from the published literature (figure 1-left) did not match.

### Intensity of testing vs. intensity of treatment

**Figure 2:**
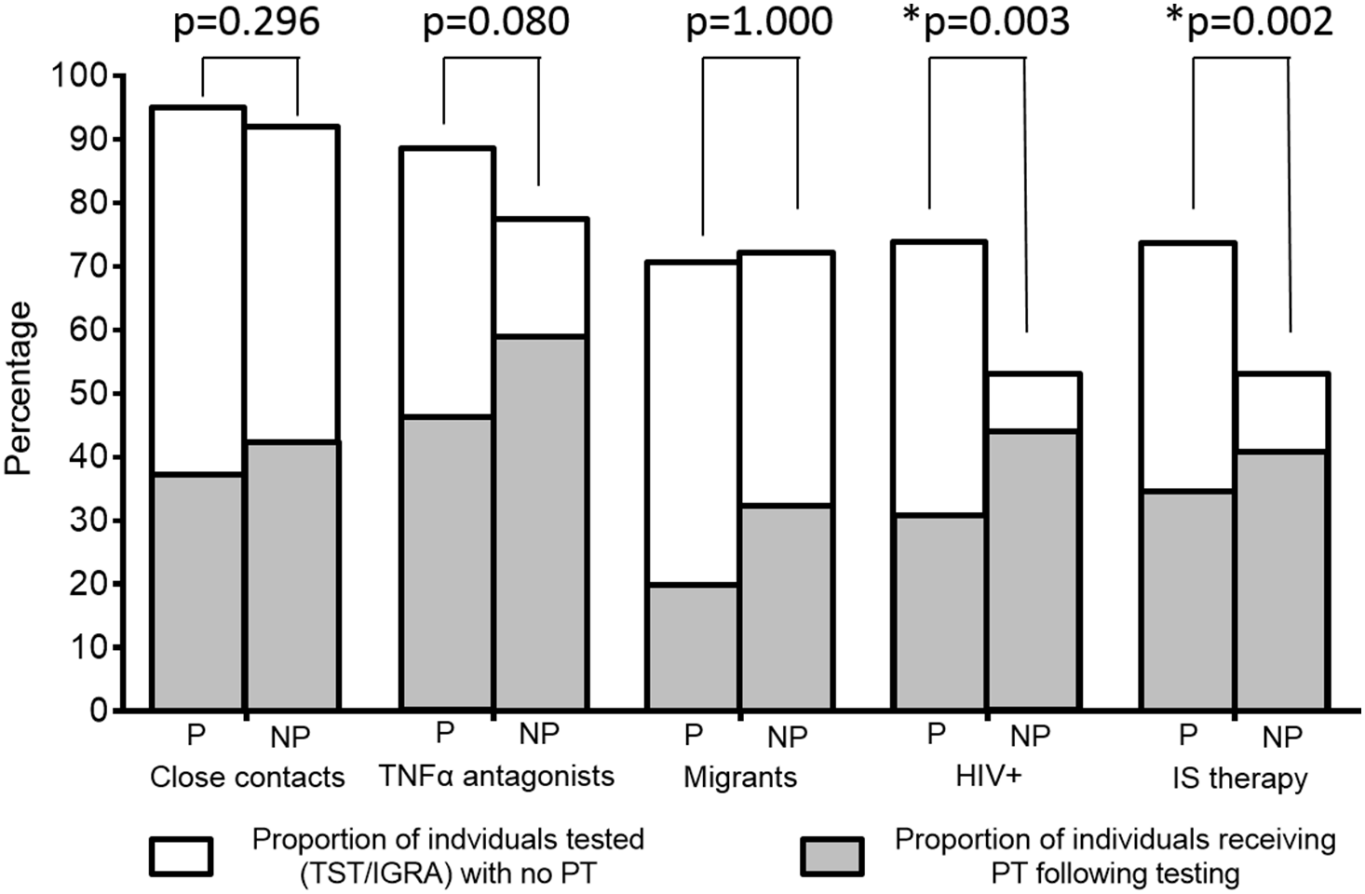
Rate of performed tests (IGRA/TST) and preventive treatment offered in the case of a positive test result in risk groups among pulmonologists and non-pulmonologists involved in TB prevention in Germany. P – pulmonologists; NP – non-pulmonologists; TNFα – tumor necrosis factor α; IS therapy – immunosuppressive therapy, IGRA – interferon gamma release assay; TST – tuberculin skin test, PT – preventive chemotherapy.

The majority of physicians recommend immunodiagnostic testing for LTBI (figure 2). For close contacts of patients with TB 94% of pulmonologist and 91% of non-pulmonologists suggest testing. Similar results can also be observed for other risk groups such as patients undergoing TNF-antagonist therapy or migrants. However, there is a substantial discrepancy between the intention to test and the intention to treat. Physicians in Germany intend to treat only 50% of individuals undergoing TNF-antagonists therapy, 40% of close contacts of patients with TB, 36% of individuals with HIV-infection and 25% of migrants with a positive result in the TST and/or IGRA. Remarkably, for most risk groups no difference can be identified in the attitudes of pulmonologists vs. non-pulmonologists towards the intention to treat. The only significant difference was found towards individuals with HIV-infection and those undergoing immunosuppressive therapy.

### Choice of preventive treatment of latent infection with *M. tuberculosis*

Physicians were able to choose between five different TB preventive treatment regimens or enter manually a treatment regime of their choice. Three hundred and sixty-two of 510 (71%) physicians answered the questions. The preferred preventive treatment is isoniazid for 9 months (n = 194; 54.4%), followed by isoniazid for 6 months (n = 85; 23.5%) and treatment with the combination of daily treatment with isoniazid and rifampicin for 3 months (n = 46; 12.7%). Fifteen physicians (4.1%) favored daily treatment with isoniazid monotherapy for 12 months and 5 physicians (1.4%) daily treatment with rifampicin monotherapy for 4 months. Fourteen physicians (3.9%) entered alternative treatment options.

### Attitudes of decision makers towards TB prevention

Participants had the opportunity to express their attitudes by answering nine questions (q1–q9) concerning TB prevention and treatment (table 3). While 81.1% of the physicians agree that immunodiagnostic testing for LTBI in risk groups and treatment of individuals with a positive test result is an efficient method of TB prevention, more than 30% disagree to the principle “intention to test is intention to treat!”. The physicians who responded in the survey agreed that more than 40% of physicians and more than 55% of patients have no insight into the efficacy of preventive treatment. A large proportion of physicians (58.4%) and patients (69.0%) are thought to be hesitant to enter treatment for fear of adverse drug events.

**Table 3:** Attitudes of German physicians involved in LTBI testing and/or the decision for the initiation of tuberculosis preventive chemotherapy.

Questions towards decision makers concerning tuberculosis prevention		Agreement			Chi-Square Test p-value
		Pulmonologists	Non Pulmonologists	All	
q1	Testing persons at risk with TST/IGRA and treatingindividuals with a positive test result isan efficient method of prevention.	78.3% (n = 148)	84.1% (n = 143)	81.1% (n = 291)	0.179
q2	“Intention to test is intention to treat!”	67.4% (n = 128)	71.6% (n = 121)	69.4% (n = 249)	0.423
q3	A risk analysis through TST and/orIGRA should be performed with allindividuals belonging to a risk group	66.8% (n = 125)	68.3% (n = 114)	67.5% (n = 239)	0.821
q4	“Tuberculosis is on the decline inGermany and prevention is not necessary anymore!”	14.9% (n = 28)	14.4% (n = 24)	14.7% (n = 52)	1.000
q5	“A positive test result (TST/IGRA)has no significance to me!”	19.4% (n = 36)	14.3% (n = 24)	16.9% (n = 60)	0.256
q6	Physicians have no insight into theefficacy of preventive treatment	50.0% (n = 94)	30.5% (n = 50)	40.9% (n = 144)	0.0002*
q7	Physicians avoid to administerpreventive treatment for the risksof side effects	61.9% (n = 117)	54.5% (n = 91)	58.4% (n = 208)	0.163
q8	Patients have no insight into theefficacy of preventive treatment	56.8% (n = 108)	54.5% (n = 91)	55.8% (n = 199)	0.671
q9	Patients hesitate to enter preventivetreatment for the fear of side effects	70.2% (n = 134)	67.7% (n = 113)	69% (n = 247)	0.648

### Measures to improve TB prevention

Finally, physicians were asked to prioritize three statements concerning optimizing TB prevention. Most physicians (n = 173; 57.5%) favored improvements in the diagnostics of LTBI, while there was an equal proportion of physicians that favored a stronger efficacy of preventive treatment regimens (n = 129; 41%) or improvements to shorten the duration of preventive chemotherapy (n = 117; 39%).

## Discussion

TB has become a rare disease in Germany and most other Western European Countries. As the World Health Organization (WHO) and the European Center for Disease Prevention and Control (ECDC) now aim for TB elimination [11], [12], prevention of TB will focus especially on risk groups. However, currently available tests are poor prognostic markers for the identification of individuals who will develop TB in the future and the definitions of “risk groups for TB” are not universally applicable [13], [14].

We evaluated the knowledge about TB risk factors and attitudes towards TB prevention among physicians involved in TB prevention and care in Germany. The key findings of this study are a surprisingly low proportion of individuals with LTBI belonging to classical risk groups for TB receiving preventive therapy and substantial gaps in the knowledge on the risk for TB in a country of low TB incidence resulting in uncertainties and non-stringent management of TB prevention.

Pulmonologists are more likely to note that physicians have no insight into the efficacy of preventive therapy than non-pulmonologists. This is likely due to the better knowledge of pulmonologists on tuberculosis, compared to non-pulmonologists. Although pulmonologists are more motivated to test HIV-infected individuals and other immunocompromised hosts for LTBI compared to non-pulmonologists, and they are better aware of the gaps in TB control in Germany, stringency to provide preventive chemotherapy for individuals with positive test results is lacking in all groups of professionals.

In the absence of available data from Germany, national recommendations for TB contact tracing [7] report that people living with HIV-infection (PLWH) have a risk of developing active TB of 35–162 per 1000 person-years. However these data originate from studies conducted before the advent of modern antiretroviral therapies (ART) [15] and refer to high prevalence countries of TB, where *M. tuberculosis* exposure for PLWH is much higher than in Germany. Results from the Swiss HIV cohort reported a lower incidence of active TB of 16 per 1000 person-years in TST positive individuals in the absence of preventive therapy [16]. Furthermore, the country of origin was of substantial importance for the risk of TB in that study. In Switzerland the number of PLWH with a positive TST or IGRA test result who needed to be treated to prevent a case of TB was 4 times higher for migrants from high incidence countries of TB compared to individuals originating from low incidence countries of TB [16]. The lower risk of PLWH for developing active TB in low-prevalence settings is likely related to a decreased risk of *M. tuberculosis* transmission from individuals with active disease, while in high-incidence countries the risk of *M. tuberculosis* exposure is considerably greater.

Additionally, in a low incidence setting, ART initiation leads to a 44–56% risk reduction of active TB [16], [17]. PLWH predominantly receive their care from specialized outpatient clinics or private practitioners in Germany. More than 80% of PLWH in the country have suppressed levels of viral replication on ART [18] and active TB in these patients has become a very rare opportunistic infection [19] even in the absence of preventive therapy. Persistent viral replication was also associated with a higher risk of developing TB in a large French cohort of PLWH [20]. Another recent study reporting on the German HIV cohort describes an important decrease in TB occurrence after ART initiation. The authors also suggest that country of origin and the degree of immunosuppression are also associated with the risk of developing TB. Preventive therapy was given to only a very small fraction of the population and the authors suggest a differentiated approach in ascertaining the risk of future TB and therefore the indication LTBI screening and preventive therapy within this population [21]. It is likely that because of the personal experience of physicians caring for PLWH in Germany caretakers regularly evaluate only two thirds of PLWH for LTBI and only one third of those with a positive TST or IGRA result receives preventive therapy in the present study.

Similarly, the current recommendations for TB contact tracing in Germany describe a relative risk of 37–74 for the development of TB in solid organ transplant recipients. However, in a large retrospective cohort study of lung transplant patients from Germany totaling over 7000 person-years of follow-up, only 5 patients were diagnosed with active TB corresponding to relative risk of 7.5–10 times lower than indicated, in this population [22]. In our survey, only one third of physicians indicated that they offered preventive chemotherapy to immuno-suppressed patients, e.g. solid organ transplant recipients.

Until recently, one of the dogmas of immunodiagnostic testing by TST and IGRAs in individuals from risk groups was “intention to test is intention to treat” [23]. Healthcare workers (HCW) with a positive IGRA test result in countries of low TB incidence were thought to be at risk for the development of TB and were offered preventive chemotherapy. However, there is substantial within-subject variability on serial testing [24] and positive IGRA test results revert to negative in a substantial proportion of HCW in the absence of preventive chemotherapy [25]. In a recent study from North America with more than nine thousand healthcare workers of which 1223 had positive IGRA test results, one third had reversion to a negative test result on follow-up and none of the HCW developed tuberculosis, in the absence of preventive chemotherapy [26]. Therefore the benefit of preventive therapy in HCW with positive IGRA results in the absence of a documented recent exposure to an active case or additional risk factors for TB is unclear. Preventive treatment of HCW with evidence of LTBI without evidence of recent exposure is no more recommended in Germany but can be considered in cases who had a documented contact with an index case, similar to contacts in the general population [27].

National recommendations for TB contact tracing also report that the risk of patients with silicosis to be 30 times elevated [7]. In a study on 118 retired coal miners in Germany, almost 40% with silicosis, approximately 50% had a positive IGRA test result. None of the 90 individuals who were evaluated after 2 years in follow-up developed active tuberculosis in the absence of preventive chemotherapy [28].

More than one third of close contacts of patients with contagious TB can be identified as having LTBI [29]. In the absence of preventive chemotherapy the risk of close contacts with positive TST results to progress to active disease has been estimated around 2–6% during the first 2–3 years after exposure [13], [30]. However two studies report progression rates to active TB of 12–13% in close contacts with positive IGRA test results not receiving preventive chemotherapy [10], [31]. It is thus possible, that close contacts are the group with the highest risk for the progression to TB in Germany. Other studies also suggest that the risk in contacts of TB patients and particularly in household contacts is underestimated [32]. Given the fact that LTBI testing of close TB contacts is mandatory in Germany according to the Infection Protection Act the low acceptance rate documented in this survey (Fig. 2), confirming recent observations, is surprising [9], [23].

In order to improve TB prevention and to achieve the goal of TB elimination in countries of low TB incidence the indication for preventive chemotherapy should be made on a risk assessment-based approach where the need to screen individuals is prioritised on the basis of the intensity of exposure and susceptibility of individuals for *M. tuberculosis* infection [3]. Additionally, due to the low positive predictive value, LTBI-testing should not be directed at individuals with a low risk of active TB in whom the risks of preventive chemotherapy may outweigh its benefits. It has been suggested that over 95% of individuals with a positive IGRA or TST do not develop active TB during follow-up further supporting targeted testing [33].

Rather surprisingly we found that physicians in Germany did not rank migrants among the groups with a high risk for TB, although it is recognized that individuals coming from high TB prevalence countries who are latently infected might have an increased risk of active TB of more than 13-fold in comparison with migrants without LTBI [34]. Other studies also report insufficient testing coverage of migrants for LTBI [35].

Even though preventive therapy is highly effective in selected populations, acceptance and adherence to a prolonged treatment are less than optimal and adverse effects, although rare, can occur in a small proportion of individuals. Mistrust against preventive strategies might explain the suboptimal acceptance rates for preventive therapy among both patients and prescribing physicians with even lower acceptance rates being recorded among HCW. On the other hand, the long duration of therapy might lead to poor adherence with fewer than half of the individuals started on preventive therapy completing the entire course according to one study [36]. Consequently in the low-prevalence setting, the decision for preventive treatment should be based on an individualized risk-benefit assessment [37], [38].

This study has several limitations. Physicians’ behavior was not directly analyzed but attitude of behavior was evaluated. It is possible, that actual behavior differs from the stated attitudes. Although the survey was distributed among a large group of decision makers and caretakers of TB patients in Germany from the public health care sector and different clinical areas and 510 physicians completed the online survey, the response rate of the electronic questionnaire was only approximately 20%. Due to the anonymous nature of this web-based survey, no information was available on the physicians who responded or did not respond to the invitation to participate in the study. Consequently, sampling bias cannot be excluded and generalization of the results has to be made with caution. Despite these limitations, this is the largest survey of physicians’ attitude towards TB prevention in Germany to date and the results from this survey reflect actual data on acceptance of preventive chemotherapy in this country.

In conclusion, we found great uncertainty about risk factors for tuberculosis among physicians in Germany likely leading to non-stringent behavior in TB prevention. TB prevention could be improved if the definition of TB “risk groups” for LTBI screening and preventive chemotherapy will be re-classified according to data applying to local situations. Immunodiagnostic testing should be limited to risk groups in which a positive test result is associated with a significantly increased risk for developing TB in the future and significant risk reduction can be achieved by preventive chemotherapies. This will require regional and national surveys rather than applying information from high TB prevalence countries to countries of low TB prevalence and vice versa. This approach could lead to more consequent initiation of preventive therapy following a positive test and avoid unnecessary testing and treatment.
